# Application of a next-generation sequencing (NGS) panel in newborn screening efficiently identifies inborn disorders of neonates

**DOI:** 10.1186/s13023-022-02231-x

**Published:** 2022-02-21

**Authors:** Xinwen Huang, Dingwen Wu, Lin Zhu, Wenjun Wang, Rulai Yang, Jianbin Yang, Qunyan He, Bingquan Zhu, Ying You, Rui Xiao, Zhengyan Zhao

**Affiliations:** 1 Department of Genetics and Metabolism, Children’s Hospital of Zhejiang University School of Medicine, National Clinical Research Center for Child Health, Hangzhou, People’s Republic of China; 2 Hangzhou Biosan Clinical Laboratory Co. Ltd, 859 Shixiang West Road, Hangzhou, Zhejiang Province People’s Republic of China; 3 Zhejiang Biosan Biochemical Technologies Co. Ltd, 859 Shixiang West Rd, Hangzhou, 310007 Zhejiang Province People’s Republic of China; 4 Zhejiang Neonatal Screening Center, Department of Genetics and Metabolism, Children’s Hospital of Zhejiang University School of Medicine, National Clinical Research Center for Child Health, Hangzhou, China; 5 Department of Child Healthcare, Children’s Hospital of Zhejiang University School of Medicine, National Clinical Research Center for Child Health, Hangzhou, People’s Republic of China; 6 Department of PediatricsChildren’s Hospital of Zhejiang University School of Medicine, National Clinical Research Center for Child Health, 3333 Binsheng Rd, Hangzhou, 310052 Zhejiang Province People’s Republic of China

**Keywords:** Newborn genetic screening, Inherited disorders, Multiple PCR, Gene mutation

## Abstract

**Background:**

Newborn screening (NBS) has been implemented for neonatal inborn disorders using various technology platforms, but false-positive and false-negative results are still common. In addition, target diseases of NBS are limited by suitable biomarkers. Here we sought to assess the feasibility of further improving the screening using next-generation sequencing technology.

**Methods:**

We designed a newborn genetic sequencing (NBGS) panel based on multiplex PCR and next generation sequencing to analyze 134 genes of 74 inborn disorders, that were validated in 287 samples with previously known mutations. A retrospective cohort of 4986 newborns was analyzed and compared with the biochemical results to evaluate the performance of this panel.

**Results:**

The accuracy of the panel was 99.65% with all samples, and 154 mutations from 287 samples were 100% detected. In 4986 newborns, a total of 113 newborns were detected with biallelic or hemizygous mutations, of which 36 newborns were positive for the same disorder by both NBGS and conventional NBS (C-NBS) and 77 individuals were NBGS positive/C-NBS negative. Importantly, 4 of the 77 newborns were diagnosed currently including 1 newborn with methylmalonic acidemia, 1 newborn with primary systemic carnitine deficiency and 2 newborns with Wilson’s disease. A total of 1326 newborns were found to be carriers with an overall carrier rate of 26.6%.

**Conclusion:**

Analysis based on next generation sequencing could effectively identify neonates affected with more congenital disorders. Combined with C-NBS, this approach may improve the early and accurate identification of neonates with inborn disorders. Our study lays the foundation for prospective studies and for implementing NGS-based analysis in NBS.

**Supplementary Information:**

The online version contains supplementary material available at 10.1186/s13023-022-02231-x.

## Introduction

Newborn screening (NBS) is one of the most successful public health initiatives for preventing disability and death. Since the first NBS was started in the 1960s to test for phenylketonuria (PKU), diseases included in NBS have been expanded extensively [1, 2]. The introduction of tandem mass spectrometry (MS/MS) is one of the most important advances in NBS, because it allows more than 40 metabolic disorders to be detected from dried blood spot (DBS) samples [3, 4].

Although previous screening methods featured simple, rapid, and convenient procedures, limitations also exist because metabolites or enzymatic activities are often influenced by multiple factors [5, 6]. Some disorders, such as hearling loss, do not develop evident symptoms during the regular NBS time window; therefore, these disorders could not be detected readily by current NBS methods [7]. Analysis of disease-associated DNA molecules or genes could be a great supplemental technology for conventional screening, particularly for the disorders without specific metabolites or biomarkers such as severe combined immune deficiency (SCID) and spinal muscular atrophy (SMA) [8–10].

A variety of DNA-based techniques have been applied in NBS of specific disorders. Recent development and rapid implementation of next-generation sequencing (NGS) in the molecular diagnosis of genetic disorders provide further options and opportunities for DNA-based NBS, as exemplifed by NGS-based genetic analysis as a regular diagnostic work-up following an abnormal NBS result [4, 11]. For disorders with clearly defined pathogenic variations, genetic screening can be used in first-tier tests of SCID [8, 12], XLA [9], SMA [10, 13], fragile X syndrome [14, 15] and hearing loss [16]. It has been reported that genetic testing as second-tier analyses improves the diagnostic specificity of CF [17], MMA [18], DMD [19] and NICCD [20]. In 2013, four genetic screening projects (BabySeq/NBSeq/NC NEXUS/STATseq) based on NGS to explore the clinical utility of whole exome sequencing (WES) and whole genome sequencing (WGS) were implemented in NBS. Although genome-wide genetic screening provides information for significantly more genetic disorders, the interpretation and reporting of genomic variants remain challenging, such as the heteroplasmy of mitochondrial variants and the unknown significance (VUS) variants. In addition, WES and WGS are still relatively expensive and the amount of DNA from dried blood is limited for sequencing library preparation [21]. Consequently, WES/WGS were not considered suitable for first-tier testing, but could be of merit as second-tier testing according to the programs [11, 22–24]. A more efficient and less expensive approach is warranted for sequence analysis of DNA from DBS to screen for congenital disorders in neonates.

In the present study we designed a newborn genetic sequencing (NBGS) panel based on multiplex PCR to analyze sequences of 134 genes of 74 neonatal inborn disorders that were validated with 287 samples that have molecular diagnosis previously. Furthermore, retrospective studies were performed with 4986 archived DBS for exploring a potential screening strategy that combines conventional NBS (C-NBS) with genetic screening of neonatal inborn disorders, as well as increasing the capacity to screen out diseases not contained in C-NBS.

## Materials and methods

### Study subjects

A total of 287 samples with molecular diagnoses from previous genetic analyses were enrolled in the validation cohort to validate the performance of the NBGS panel. The retrospective cohort includes two groups of 4986 samples in total, from neonates born between January 2017 and December 2019, who received C-NBS, including inherited metabolic disease screening by MS/MS methods, and congenital hypothyroidism (CH) and glucose-6-phosphate dehydrogenase deficiency (G6PDD) screening by biochemical methods. Of the 4986 samples, group 1 of 2973 were randomly selected from those with a negative initial C-NBS results, and group 2 of 2013 were from samples with a positive result in the initial C-NBS, which received repeat MS/MS analysis. All 4986 samples were analyzed with NBGS, of which 5 samples were excluded from data analysis due to low quality of NGS data and 4981 NGS result were included in the following analysis. In group 2, 53 newborns were C-NBS true positive as confirmed by repeat C-NBS and by clinical manifestation, 8 samples were lost to follow-up, 5 infants with unexplained death, and 1947 were negative by repeat C-NBS and were denoted as MS/MS false-positive cases. The potential capacity of NBGS to reduce C-NBS false-positives and C-NBS false-negatives was estimated in 1947 MS/MS false-positive newborns and the remaining newborns that were undiagnosed clinically, respectively. Also, the value of NBGS to screen disorders out of C-NBS was evaluated in these newborns. The C-NBS result, hearing screening outcome and clinical profiling including newborn gestational age (GA, in weeks), birth weight (BW, in grams) were collected from all newborns. This study was approved by the institutional review board of the ethics committee in Children’s Hospital, Zhejiang University School of Medicine.

### Selection of disorders, genes and variants

Seventy four inborn disorders were included in this panel based on the Secretary’s Advisory Committee on Heritable Disorders in Newborns and Children and the Wilson and Jungner criteria [25, 26], disorders that can be detected by biochemical test were selected with consideration of Recommended Uniform Screening Panel (RUSP) recommendation, and disease with high incidences but cannot be screened by C-NBS in China were also enrolled. The disorders in this panel consist of inherited metabolic diseases screened by MS/MS, G6PD, CH and genetic diseases with high incidences and severe impact on children’s health (Additional file 1: Table S1), including 39 inborn metabolic disorders screened by MS/MS and 35 disorders of skeletal system diseases, hematological system diseases, lysosomal storage disease, etc., of which 33 disorders are not covered by C-NBS. A total of 134 genes were included according to relative prevelance in associated 74 disorders. Known pathogenic variants in 134 genes (Additional file 2: Table S2) were selected as targets of the panel according to one of the following criteria: (1) high frequency in Chinese, Eastern Asian or Asian population; (2) common pathogenic variants in databases including ClinVar and ClinGen; (3) loss of function (LOF) variants in Asian population (≥ 10 allele count in Gnomad) (4) high frequency in local databases. A multiplex PCR panel was designed with each selected variant covered by at least one amplicon.

### Library construction and sequence analysis

Genomic DNA were extracted from dried blood spots by Nucleic Acid Automatic Extraction System (Bioer, China). DNA libraries were prepared based on multiplex PCR by using the SLIMamp (StemLoop Inhibition Mediated amplification) method [27]. High-throughput sequencing was performed with Illumina NextSeq 500 according to the manufacturer’s instruction.

### Bioinformatic analysis

Raw sequencing data were processed using general procedures. Low-quality sequencing reads were removed and the reads were mapped to the NCBI human reference genome (hg19/GRCh37). After variant calling and annotation, potential pathogenic variants were kept for further evaluation. In addition to the pre-selected pathogenic variants, pathogenicity of all other variants were evaluated according to ACMG sequence variant interpretation guideline [28]. Only pathogenic and likely pathogenic variants were considered for reporting.

### IEM panel

Inborn errors of metabolism (IEM) panel is an in-house targeted NGS panel of 86 genes (*PAH*, *PTS*, *MUT*, *SLC22A5*, etc.) associated with inherited metabolic disorders. Coding exons of target genes were captured using an Agilent High Sensitivity DNA Kit (Agilent, Santa Clara, CA, USA). Libraries generated from enriched DNA were sequenced using the Illumina NextSeq 500 platform (Illumina Inc., San Diego, CA, USA) in paired-end mode.

### Statistical analysis

The comparison of the biochemical index, GA and BW of different groups was done by analysis of variance (ANOVA), and statistical analysis between any two groups was performed using a t-test (two-tailed).

## Result

### Evaluation of the performance of NBGS panel

In the validation cohort of 287 samples, 155 mutations of 35 genes were covered in the NBGS. All mutations in 35 genes were identified by NBGS except for one variant in the *MMACHC* gene (c.482G > A) in one sample due to low data quality. Overall, the accuracy of the NBGS was 99.65% (286/287). A total of 33 mutations were detected in the *PAH* gene, followed by 18 mutations in the *SLC22A5* gene and 10 mutations in the *MMUT* gene (Additional file 1: Fig. S1).

### Newborn genetic screening of the retrospective cohort

**Fig. 1:**
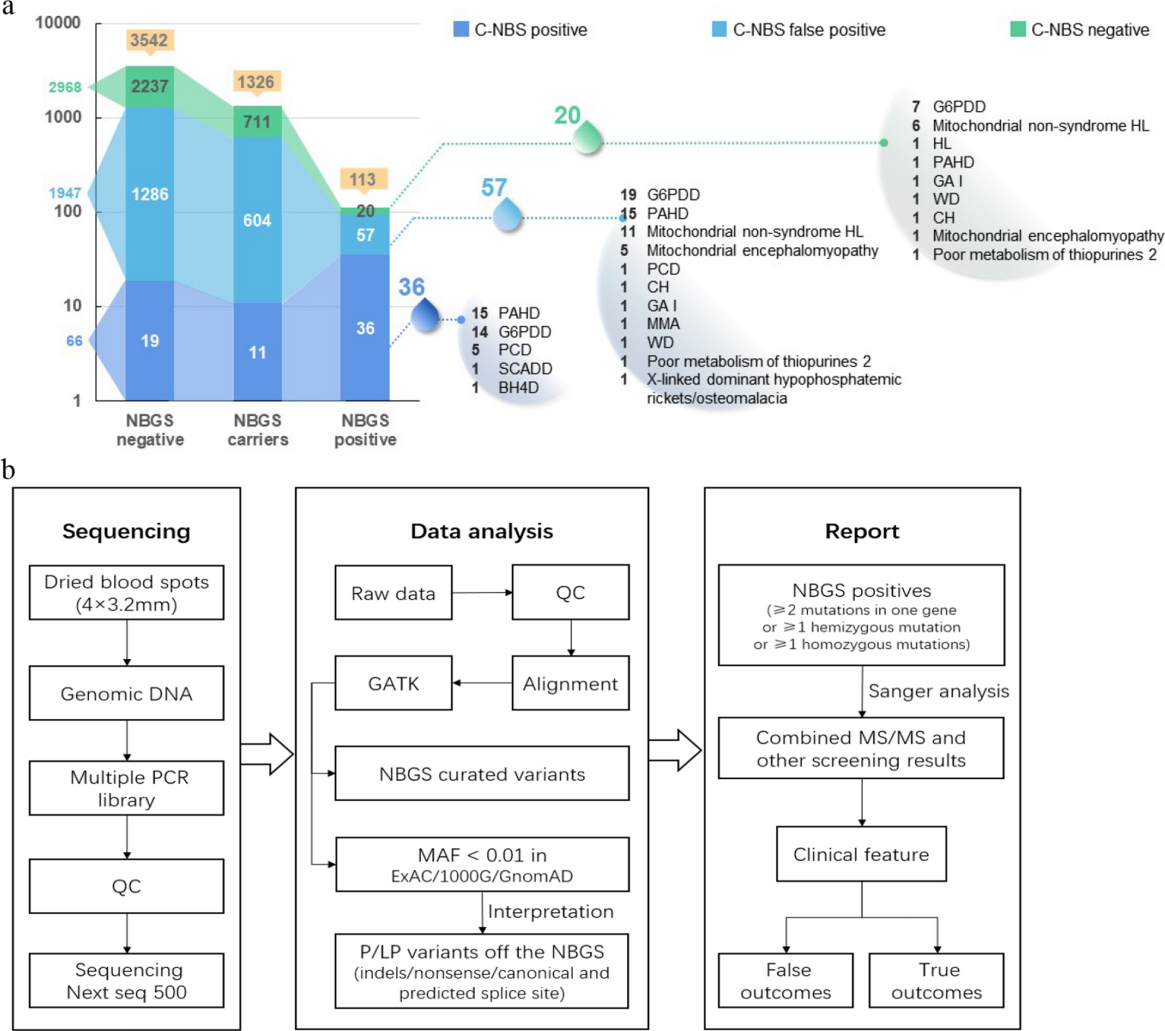
Newborn genetic screening of the retrospective cohort.) Comparison of C-NBS and NBGS result in 4981 recruited newborns; **b** The work flow of NBGS panel

A cohort of 4986 newborns were subjected to NBGS analysis (5 samples with low-quality data were excluded) and compared with C-NBS result (Fig. 1a). The work flow of NBGS was shown in Fig. 1b. The characteristics of all newborns were shown in Table 1, and a small number of preterms born at 32–37 weeks were included in our study, which make up the majority of newborns with low birth weight (1500–2500 g).

**Table 1:** The demographic characteristic of all newborns in this study

	N (%)/Median/Mean
*Gender*	
Male	2635 (52.8%)
Female	2351 (47.2%)
*Birth weight*	
Mean	3264 ± 539* g*
Median	3330 g
< 1000 g	6 (0.1%)
1000–1500 g	64 (1.3%)
1500–2500 g	301 (6.1%)
2500–4000 g	4300 (86.2%)
> 4000 g	315 (6.3%)
*Gestational age*	
Mean	38 ± 2.5 weeks
Median	39 weeks
< 28 weeks	9 (0.2%)
28–32 weeks	101 (2%)
32–37 weeks	376 (7.5%)
37–42 weeks	4494 (90.2%)
> 42 weeks	6 (0.1%)
C-NBS true positive	53
Lost to follow-up	7
Unknown death	6
C-NBS false positive	1947
C-NBS negative	2973
Total	4986

**Fig. 2:**
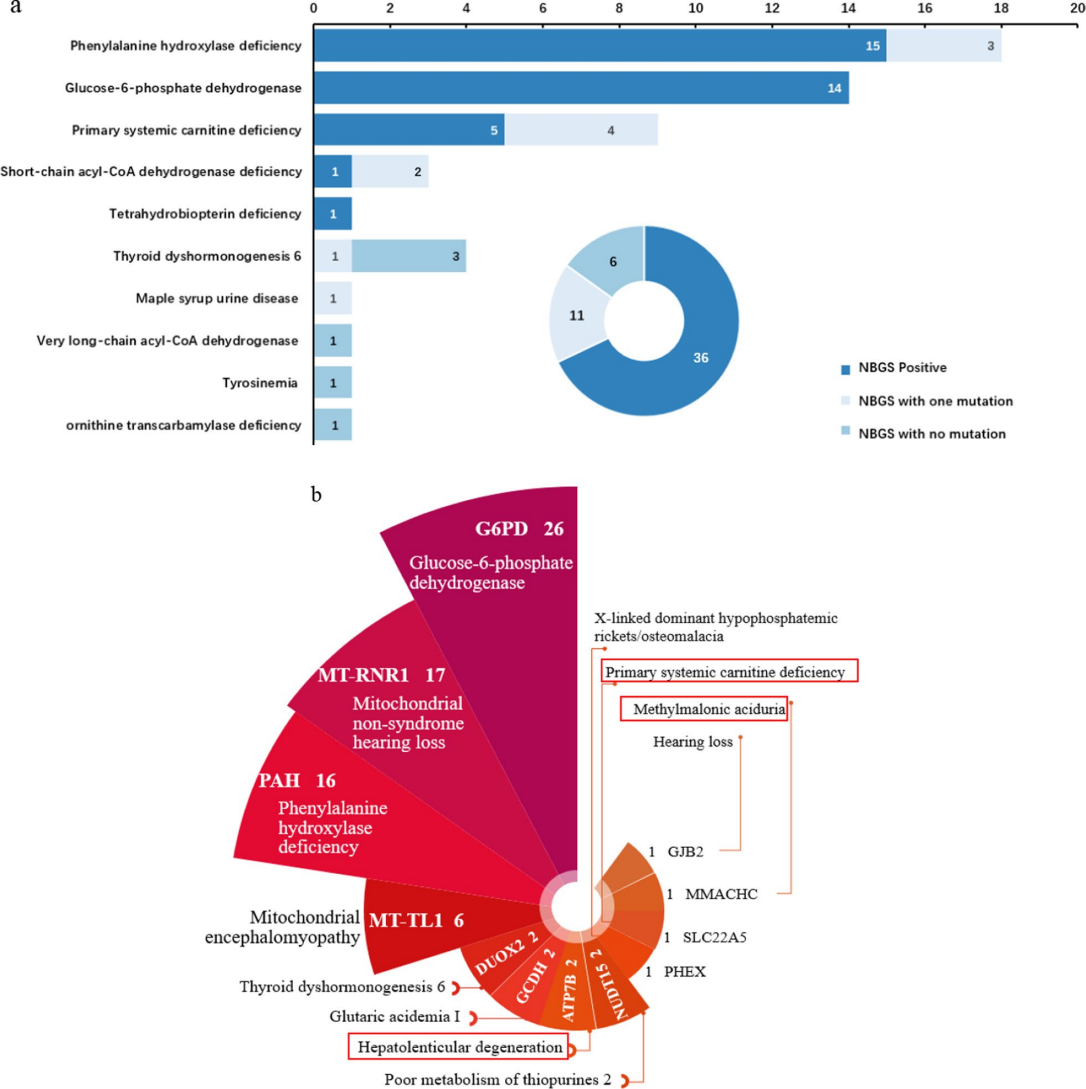
Diagnosed and C-NBS negative newborns identified by NBGS. **a** Genetic analysis of the 53 diagnosed newborns by NBGS; **b** The distribution of 77 C-NBS negative newborns that detected positive by NBGS panel (the red boxes represent diagnosed newborns)

In the 53 C-NBS true positive subjects, who received positive repeat C-NBS results, 36 cases were identified as positive by NBGS, and the detection rate was 68% (36/53) (Fig. 1a, Fig. 2a), the biochemical results and genotypes were shown in Table 2. Importantly, all newborns with positive C-NBS results indicating G6PDD and tetrahydrobiopterin deficiency (BH4D) were confirmed by NBGS. For seventeen NBGS negative samples, only one mutation related to the diagnosed disease were detected in 11 newborns whereas no mutations were detected in 6 subjests (Fig. 2a). Subsequently, the NBGS negative samples were subjected to IEM panel for further analysis and biallelic or hemizygous mutations in the disorder-related genes were identified in 13 subjects (Additional file 1: Table S3).

**Table 2:** The C-NBS true positive newborns identified positive by NBGS

NO	Conditions (Gene)	Biochemical range	Positive rule	NBGS result	Cases
1	PAHD (*PAH*)	PHE: 120–769 μmol/L PHE/TYR: 1.2–12	PHE > 100 μmol/L, PHE/TYR > 1.2	[c.1068C > A/c.505C > A]; [c.688G > A/c.688G > A]; [c.728G > A/c.728G > A]; [c.722delG/c.466G > C]; [c.1216A > G/c.8C > T]; [c.721C > T/c.721C > T]; [c.194 T > C/c.158G > A]; [c.975C > G/c.158G > A]; [c.1068C > A/c.158G > A]; [c.1162G > A/c.158G > A]; [c.331C > T/c.158G > A]; 2* [c.728G > A/c.158G > A]; [c.1252A > C/c.158G > A]; [c.838G > A/c.1123C > G];	15
2	G6PDD (*G6PD*)	G6PD/6PGD: 0.3–0.9	G6PD/6PGD < 1.0	**Male** 5* [c.1466G > T];3* [c.1478G > A] 2* [c.1114C > T]; 2* [c.185A > G]; **Female** [c.185A > G]; [c.1388G > A]	14
3	PCD (*SLC22A5*)	C0: 7–7.3 μmol/L	C0 < 9.5 μmol/L	2* [c.760C > T/c.1400C > G]; [338G > A/c.1400C > G]; [c.428C > T/c.1400C > G]; [c.497 + 1G > T/c.760C > T]	5
4	SCADD (*ACADS*)	C4 = 1.14 μmol/L	C4 > 0.7 μmol/L	c.164C > T/c.1130C > T	1
5	Tetrahydrobiopterin deficiency (*PTS*)	PHE = 318 μmol/L PHE/TYR = 4.2	PHE > 100 μmol/L, PHE/TYR > 1.2	c.317C > T/c.84-291A > G	1
Total				36	

Moreover, 13 newborns including 7 lost to follow-up infants and 6 unknown death cases were suspected positive by C-NBS but were identified as negative by NBGS. Further analysis was performed by IEM panel, and 2 of the lost to follow-up subgroup were identified biallelic mutations in *CPS1* and *ACADSB* gene in two subjects with clinical diagnosis of carbamoyl phosphate synthetase I deficiency and 2-methylbutyryl-CoA dehydrogenase deficiency respectively (Additional file 1: Table S4).

A total of 77 newborns from C-NBS false-positive (57/1947, 2.9%) and C-NBS negative (20/2973, 0.7%) group were identified as positive by NBGS (Fig. 2b): 48 of 77 newborns suggested to have metabolic disorders by NBGS (Table 3), including 26 female with normal biochemical results with heterozygous G6PD mutations, 16 newborns with elevated primary MS/MS screening results suggestive of mild hyperhenylalaninemia with biallelic mutations in the *PAH* gene, 2 newborns with biallelic mutations in the *GCDH* gene for GA I, 2 CH also identified positive by NBGS and the TSH concentration was normal after followed up, and significantly 1 PCD and 1 MMA were identified positive by NBGS, which was consistent with the followed-up biochemical results, indicating a false-negative C-NBS finding at primary screening (Table 4). The remaining 29 newborns were found to carry biallelic or hemizygous mutations causing hearing loss and 4 disorders were not included in C-NBS (mitochondrial myopathy, Wilson’s disease (WD), purine disorder, and rickets) (Additional file 1: Table S5), 2 of them with biallelic [c.2333G > A/c.3532A > G] and homozygous [c.3859G > A/c.3859G > A] mutations of *ATP7B* gene were diagnosed with WD after followed up (Table 4).

**Table 3:** The biochemical range and NBGS result of 48 C-NBS false positive and C-NBS negative newborns with variants related to metabolic disorders

NO	Conditions (Gene)	Biochemical range	Positive value	NBGS result	Cases
1	G6PDD	G6PD: 30.6–44 U/dL	G6PD** < **26 U/dL	**Female**	26
	(*G6PD*)	G6PD: 2.3–5.8 u/gHb	or G6PD** < **2.6 u/gHb	5* [c.185A > G]; [c.406C > T]; 4* [c.1376G > T];	
				8* [c.1388G > A]; [c.871G > A]; [c.1360C > T]; 2* [c.392G > T]; [c.1004C > A];3* [c.1024C > T];	
2	PAHD (*PAH*)	PHE: 102–162 μmol/L	PHE** > **100 μmol/L	[c.1256A > G/c.158G > A]; [c.721C > T/c.158G > A];	16
				[c.728G > A/c.158G > A]; [c.331C > T/c.158G > A]; [c.320A > G/c.158G > A]; 2* [c.1197A > T/c.158G > A];	
				[c.611A > G/c.158G > A]; [c.1199G > A/c.158G > A];	
				[c.964G > A/c.158G > A]; [c.1068C > A/c.158G > A];	
				[c.4421G > A/c.158G > A];2* [c.842 + 2 T > A/c.158G > A];	
				[c.721C > T/c.158G > A]; [c.208_210del/c.158G > A];	
3	GA I (*GCDH*)	C5DC + C6-OH: 0.07–0.18 μmol/L	C5DC + C6-OH > 0.4 μmol/L	2* [c.1244-2A > C/c.1261G > A]	2
4	PCD (*SLC22A5*)	C0 = 10. 5 μmol/L	C0 < 9.5 μmol/L	c.760C > T/c.1400C > G	1
5	MMA (*MMACHC*)	C3 = 3.5 μmol/L	C3** > **4 μmol/L	c.609G > A/c.617G > A	1
		C3/C2 = 0.18	C3/C2** > **0.25		
6	CH (*DUOX2*)	TSH: 2.5–5.9 μIU/mL	TSH** > **9.0 μIU/mL	[c.1588A > T/c.1588A > T]; [c.2104_2106del/c.2104_2106del]	2
Total				48

**Table 4:** Summary of clinical and genetic features of 4 diagnosed newborns by NBGS

	Case NP0467	Case NP0865	Case NP0956	Case NP0439
Gender	Male	Female	Female	Male
Age of onset	14 months	3 years old	14 months	3 years old
Biochemical result	Primary screening result:		N/A	N/A
	C3 = 3.5 μmol/L	C0 = 10.5 μmol/L		
	C3/C2 = 0.18			
	Follow-up result:			
	C3 = 5.2 μmol/L	C0 = 7.3 μmol/L		
	C3/C2 = 0.34			
Birthweight (g)	3650	2700	2900	3660
Gestational age (weeks)	39 + 2	39 + 0	38 + 3	39 + 2
Diagnosed disease	Methylmalonic aciduria	primary systemic carnitine deficiency	Wilson’s disease	Wilson’s disease
Gene	*MMACHC*	*SLC22A5*	*ATP7B*	*ATP7B*
Genotype	c.609G > A/c.617G > A	c.760C > T/c.1400C > G	c.2333G > A/c.3532A > G	c.3859G > A/c.3859G > A

N/A, not applicable

### Detection of recessive disease carriers by NBGS

**Fig. 3:**
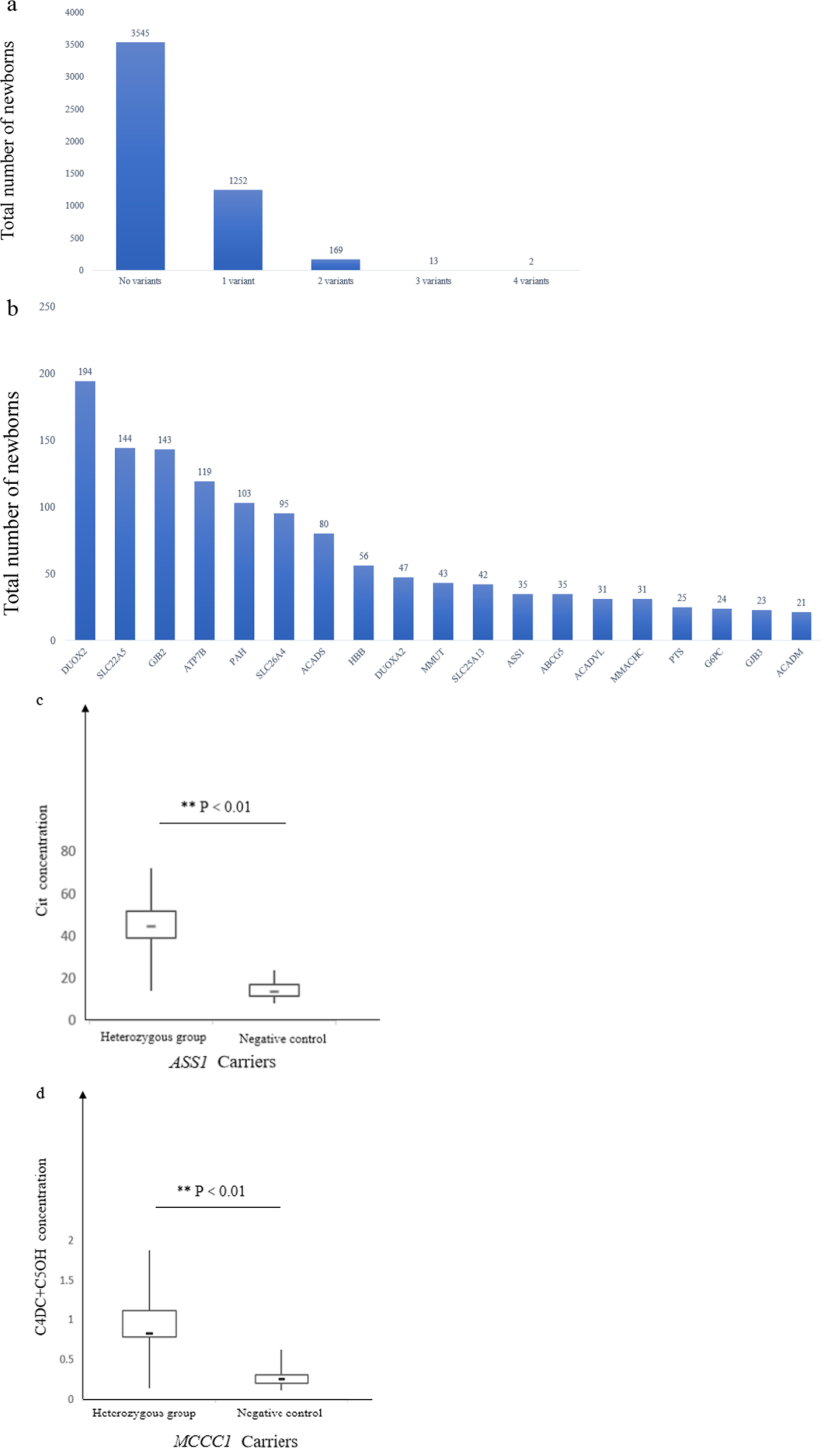
The mutation frequency in carriers and biochemical index of *ASS1* and *MCCC1* carriers. **a** The numbers of variants in carriers. **b** The distribution of high frequency gene mutations in carriers. **c** Cit concentration in *ASS1* gene mutation carriers and negative control. **d** C4DC + C5OH concentration in *MCCC1* gene mutation carriers and negative control

In the 4981 newborns, heterozygous carriers were frequently observed. Except for one individual carrying heterozygous mutations in 4 genes, most carrier newborns were found to have one mutation (Fig. 3a), whereas 184 (~ 3.7%) newborns were found to be carriers of two or more variants in different genes. In summary, a total of 1326 newborn carriers were identified through NBGS analysis with an overall carrier frequency of 26.6% (1326/4981). The top five genes with the highest carrier frequency in these newborns were *DUOX2* (14.06%), *SLC22A5* (10.54%), *GJB2* (10.47%), *ATP7B* (8.5%) and *PAH* (7.8%) (Fig. 3b). Genes with at least one carrier identified were listed in Additional file 1: Table S6, and five genes (*MTR, TG, GBA, MTHFR* and *CPT2*) of NBGS were associated with susceptibility to certain conditions that have summarized in Additional file 1: Table S7.

In addition, carrier newborns with *ASS1* and *MCCC1* mutations were noticed to have elevated levels of corresponding metabolites from C-NBS results. We compared 34 *ASS1* carriers and 17 *MCCC1* carriers with negative controls and a significant difference was found between carrier and control groups (Fig. 3c, d).

### Correlation of gestational age, birth weight, MS/MS and NBGS results

A total of 1947 newborns were false-positive by MS/MS primary screening, of which 1890 cases were identified as negative by NBGS consistent with follow-up testing. To further explore the possible factors related to inconsistent results of primary MS/MS screening and NBGS, GA and BW were analyzed in diagnosed newborns (NBGS positive), C-NBS false-positive newborns (MS/MS primary positive and NBGS negative) and control group (healthy newborns with negative C-NBS and NBGS results). The results showed that newborns in the C-NBS false positive group are associated with significantly lower BW or smaller GA, suggesting low BW and small GA might affect levels of metabolites resulting in false-positive outcomes (Additional file 1: Fig. S2a, S2b). Low BW and small GA newborns with false-positive conventional screening were further divided to subgroups based on BW (< 1000 g; 1000–1500 g; 1500–2500 g) and GA newborns (< 28 weeks, 28–32 weeks, 32–37 weeks), and no significant difference was found between each groups.

## Discussion

The compelling problems facing NBS include reducing false-positive and false-negative results, improving the positive detection rate and expanding the spectrum of screened disorders beyond current C-NBS methods [20, 29–34]. We adapted a validated genetic screening panel for newborn genetic screening to achieve the goal. Our results showed that NBGS could reliably detect targeted variants, identify newborns affected by conditions not included in current C-NBS and reduce false-positive rate by C-NBS in our recruited population that highlight the complementarity of both methods.

In C-NBS true-positive newborns, 68% (36/53) of them were identified as positive by NBGS. Several studies have demonstrated the diagnostic rates of NGS panels for IEM-affected infants were approximately 50–59% [35, 36]. However, a recent study showed that WES had an overall sensitivity of 88% for infants who screened positive by C-NBS, in which 32% of the reported variants were absent from both HGMD and ClinVar [37], consistent with the sensitivity of the NBGS panel that only reports known mutations. NBGS showed good performance in disorders with high incidence such as G6PDD (100%) and phenylalanine hydroxylase deficiency (PAHD) (83.3%), similar to the detection rates reported in other studies [32, 38, 39]. For the 17 C-NBS true-positive/NBGS negative cases, the possible reasons include: (1) CH in our study were all identified negative by NBGS, it was likely that the CH associated gene mutation identified in few patient, and genetic mechanism responsible remains to be elucidated, only 51.82% (57/110) CH patients have been reported carried biallelic mutations of 21 candidate genes [40]. Due to limited genes and hotspot mutations, false-negative result would occur in genetic screening and it was suggested that the conventional screening is more suitable for CH in this study. (2) The NBGS negative P/LP variants or disorder were not targeted in our panel. (3) The VUS variants detected by IEM panel were not included in NBGS because the unclear mechanism and interpretation of VUS was challenging for clinicians in a screening panel. As literature and clinical data evolve, the VUS may become reportable and the panel should be reassessed by then. (4) Larger copy-number variants (CNVs) could not be identified by NBGS. (5) The mechanism of genetic disease was not defined completely and the relevant genes may be undiscovered, and 11 cases were also negative after performed IEM in this study.

In the 77 C-NBS false-positive and false-negative/NBGS positive infants, G6PDD was the most prevalent condition, followed by hearing loss and PAHD. Female carriers of heterozygous *G6PD* mutations are at elevated risk of developing acute hemolysis, but could be difficult to recognize by conventional biochemical methods. It has been reported that female carriers identified by genetic screening showed mild phenotypes, which is also observed in our study [41]. Therefore, combined biochemical methods with genetic screening could improve the identification of G6PDD. Mitochondrial 12S rRNA mutations are related to aminoglycoside induced deafness [42]. The mutation frequencies of mtDNA m.1557A > G and m.1496 C > T in China were 0.20% and 0.03% respectively [43]. Genetic screening helps to identify carriers and avoid deafness caused by administration of aminoglycoside antibiotics. For all of 16 PAHD newborns identified by NBGS, the c.158G > A variant was found in trans with a pathogenic or likely pathogenic variant in the *PAH* gene. The c.158G > A variant has been reported as a pathogenic variant in hyperphenylalaninemia patients, however, this variant was also considered as "likely benign" due to high population frequency and clinical evidence [44]. The concentration of phe elevated slightly at regular intervals and no clinical intervention was performed in 16 PAHD. It seems that c.158G > A may relate to mild hyperphenylalaninemia in our enrolled population. The most common mutation of mitochondrial encephalomyopathy is m.3224A > G, however, classical presentations may be absent in some pediatric patients [45]. Because of the heteroplasmy, it is difficult to evaluate the mutation load in related tissues/organs, thereby making diagnosis more challenging. A total of six newborns were identified with carriers of m.3224A > G by NBGS, we have been followed up these cases until now, and all without any clinical manifestation, maybe it suggested that mitochondrial disorders were not suitable for newborn genetic screening.

Using NBGS, 4 cases were diagnosed with MMA, PCD and WD that had been missed by C-NBS. It has been reported that the determination of MMA by MS/MS has been affected by several factors, and one case was missed due to the concentrations of C3 and C3/C0 decrease with age [46, 47]. However, unlike metabolites, genetic screening was sufficiently accurate and specific for inborn disorders because it does not vary with age, season or maternal status. WD was not screened in C-NBS due to unavailability of specific markers. Some asymptomatic patients do not receive effective treatment before irreversible injury is present [48]. In our study, 2 newborns showed normal liver function initially but identified positive by NBGS. Both newborns were diagnosed, and the old sister of NP0439 also was determined soon. It was note-worthy that applied genetic screening to C-NBS could effectively reduce the false-negative cases of C-NBS, and also could identify an additional subgroup of patients that undetectable by C-NBS, and the turn-around time was also decreased compared with conventional method that at the time of their clinical presentation arisen, actually, the turn-around time of C-NBS methods would takes about 1–2 months, one with positive initial screening result and positive multiple retest needs other biochemical test and genetic analysis to confirm. However, the testing period of NBGS is just 10 working days and C-NBS result was also finished during the time, combined NBGS and traditional methods, the turn-around time would decreased to 2–3 weeks. Moreover, expanding the spectrum of disorders that can not be screened by MS/MS could be highly beneficial especially for those with high incidence, treatable and early diagnosis is helpful for their prognosis. In our panel, a total of 33 disorders out of C-NBS could be identified such as WD, SCID and XLA.

Recently, the carrier rate of multiple disorders had been mainly reported in carrier screening of the general population. The carriers for at least one disorder was reported to be 24% [49], as in our study also showed about 1/3 screened individuals with at least one pathogenic mutation in childhood diseases. It was challenging to provide genetic counseling for such a large amount of all infants but that is meaningful to carriers of genetic disorders to determine risk for future offspring and their parents to have additional children. In the future prospective study, we would offer an "opt-out" option for reporting carrier state when signed informed consent of NBGS testing and the related counseling. If the parents choose to report carrier result and the infant identified as carrier, we can provide genetic counseling for them. The top genes such as *PAH*, *SLC22A5*, *ACADS* and *MUT* were most frequently reported for carriers known to have high carrier frequency in reported study [46], which could verify the relative high incidence of associated inborn disorders [50, 51]. Some high-frequency mutations of *DUOX2, HBB*, *ATP7B* gene in our study were not found in previous investigations. This may be attribute to the differences in targeted population, sample size, genetic background, and detected range of mutations [47, 50]. In addition, although NBGS panel was designed to screen neonates, reporting increased risk for certain conditions may be beneficial, particularly for carrier parents. However, we would recommend additional consents and counseling for reporting these conditions since uncertainty of disease onset and reduced penetrance could cause unnecessary burden for participants and change the risk–benefit balance.

In 1890 C-NBS false-positive/NBGS negative newborns, carriers with *ASS1* and *MCCC1* mutation showed abnormal biochemical results, but all newborns with normal phenotype until now. Generally, it is unlikely that variants of carrier would impact phenotype. A previous study showed 4.3% heterozygous carriers with mild presentations such as in Hemophilia A, CH, and G6PDD [41]. Given the limited numbers in carriers with phenotype and the limited understanding of their penetrance and functional studies, the relationship of carrier status and phenotypic expression is still ambiguous [41]. Therefore, NBGS could help to exclude some false positives and to reduce follow-up time and anxiety of heterozygous carriers. Moreover, we also observed GA and BW with significant difference in false-positive newborns and control. The physiological states associated with preterm, LBW, and sick newborns and the treatments received directly affect the reliability of results for many diseases screened in public health [52–54]. Metabolite levels were influenced by birth weight and gestational age [6, 55], which might increase false positives of C-NBS but could screened out by NBGS. In low BW and small GA newborns, we have not found difference in each subgroups which maybe due to the small amount of preterm infants in our study.

In summary, our results showed that advantages of the NBGS panel as a novel newborn screening test may include: (1) analysis for more preventable and treatable disorders that are not targeted by C-NBS; (2) pre-selected and pre-curated mutations that avoid complex and cumbersome interpretation and reporting; (3) lower-cost and faster turn-around time compared with WES or capture-based NGS panels that need more complex experimental process and data analysis; (4) providing rapid diagnosis of affected newborns after screened positive by C-NBS and improving the cost-effectiveness; (5) reducing false-positive newborns identified by C-NBS. Our data suggest that it may be a more effective strategy for newborn screening to combine NBGS with C-NBS. However, the NBGS test does have limitations. Because the NBGS panel only covers pre-selected known mutations, it will inevitably miss some pathogenic variants, which may lead to false-negative results and low overall detection rates. Although capture-based NGS methods could provide almost complete coverage of coding regions of targeted genes, variants in noncoding regions such as introns and untranslated regions are usually not readily detected, as well as large copy number variations (CNV) and complex structural variations (SV). In addition, because the NBGS test only reports pathogenic and likely pathogenic variants, a significant number of rare variants, particularly missense and splicing variants, will be intepreted as VUS due to lack of reported clinical or research evidence, and thus not reported, as indicated in a recent study [37]. Nonetheless, if C-NBS and NBGS are concurrently applied, joint ananlysis of biochemical and sequence data would help optimize both tests reciprocally. Further research is needed to evaluate the clinical utility and effectiveness in a larger newborn population to explore the long-term implication of sequencing-based newborn screening.

## Conclusions

In this study, we have developed an NGS panel test based on multiplex PCR with low cost, fast turn-around-time and great specificity for newborn genetic screening of 74 disorders. Our data suggest that NBGS combined with C-NBS could improve screening efficiency, reduce the false-positive/-negative results, and also expand the spectrum of screened disorders to achieve the purpose of early detection and diagnosis.

## Supplementary Information

**Additional file 1:** Table S1, S3, S4, S5, S6, S7, Figure S1, S2.

**Additional file 2:** Table S2.

## Data Availability

The datasets analyzed during the present study are available from the corresponding author on reasonable request.

## Abbreviations

NBS: Newborn screening
MS/MS: Tandem mass spectrometry
DBS: Dried blood spots
NBGS: Newborn genetic sequencing
C-NBS: Conventional newborn screening
