# Supplementary material for: Application of a next-generation sequencing (NGS) panel in newborn screening efficiently identifies inborn disorders of neonates
Source: Orphanet J Rare Dis. 2022 Feb 21;17:66. doi: 10.1186/s13023-022-02231-x (PMC8862216; doi:10.1186/s13023-022-02231-x)
Supplement: Supplementary file 1 — Additional file 1: Table S1, S3, S4, S5, S6, S7, Figure S1, S2. [file 13023_2022_2231_MOESM1_ESM.docx]

**Additional file**

**Figures**


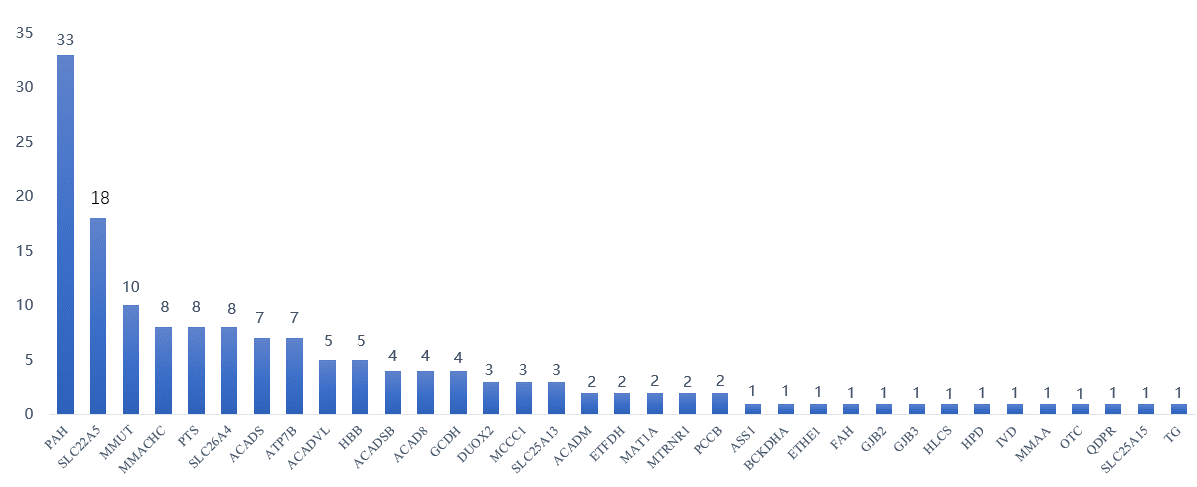


Number of mutations

Fig S1. Mutation frequencies in 35 genes from the validation cohort


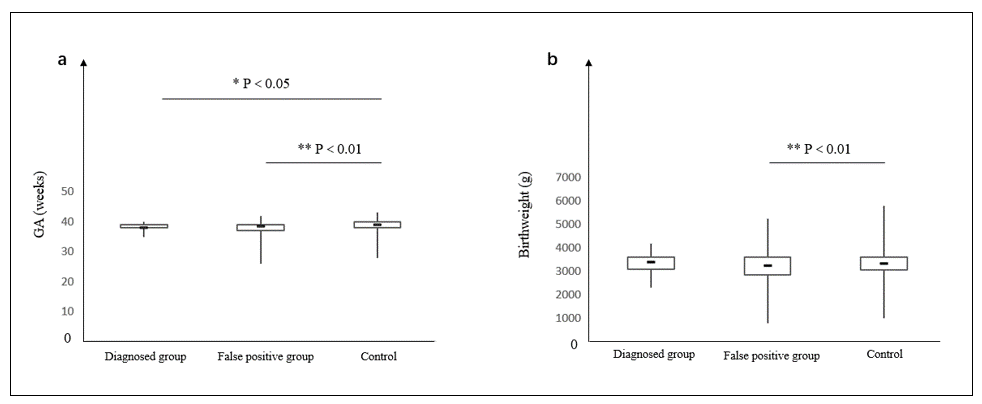


Fig. S2 Gestational age and birth weight in each group. (a) Gestational age in weeks and (b) Birth weight in grams for diagnosed newborns, false positives newborns and normal control.

**Tables**

Table S1. The list of 74 disorders included in NBGS

| NO. | Diseases |
| --- | --- |
| **Inherited metabolic diseases screened by MS/MS** | |
| 1 | Methylmalonic aciduria, MMA |
| 2 | Propionic acidemia, PA |
| 3 | Glutaric acidemia I, GA1 |
| 4 | Holocarboxylase synthetase deficiency |
| 5 | Biotinidase deficiency |
| 6 | 3-Hydroxy-3-Methylglutaryl-Coenzyme A Lyase deficiency |
| 7 | Isovaleric acidemia, IVA |
| 8 | 3-Methylcrotonyl-coenzyme A carboxylase deficiency, MCCD |
| 9 | Malonyl-CoA decarboxylase deficiency, MCD deficiency |
| 10 | 3-methylglutaconic aciduria type I |
| 11 | Ethylmalonic encephalopathy, EE |
| 12 | Isobutyryl-CoA dehydrogenase deficiency, IBDD |
| 13 | Maple syrup urine disease, MSUD |
| 14 | Tyrosinemia |
| 15 | Hyperphenylalaninemia，HPA |
| 16 | Hyperhomocysteinemia, HCY |
| 17 | Non-ketotic hyperglycinemia, NKH |
| 18 | Primary hypermethioninemia |
| 19 | Argininemia |
| 20 | Argininosuccinic aciduria |
| 21 | Carbamoyl phosphate syntetase I deficiency |
| 22 | N-acetylglutamate synthase deficiency, NAGSD |
| 23 | Ornithine transcarbamylase deficiency |
| 24 | Citrullinemia type Ⅰ, CTLN1 |
| 25 | Citrin deficiency |
| 26 | Hyperornithinemia-hyperammonemia-homocitrullinuria syndrome, HHHS |
| 27 | Ornithine transcarbamylase deficiency |
| 28 | Very long-chain acyl-CoA dehydrogenase deficiency, VLCAD |
| 29 | Long-chain 3-hydroxyl-CoA dehydrogenase deficiency, LCHAD |
| 30 | Mitochondrial trifunctional protein deficiency, MTPD |
| 31 | Short chain 3-hydroxyacyl-CoA dehydrogenase deficiency, SCHADD |
| 32 | Medium chain acyl-CoA dehydrogenase deficiency, MCADD |
| 33 | Short-chain acyl-CoA dehydrogenase deficiency, SCADD |
| 34 | Glutaric acidemia II, GA2 |
| 35 | Beta-ketothiolase deficiency, BKD |
| 36 | Carnitine palmitoyltransferase II deficiency, CPTII |
| 37 | Primary carnitine deficiency, PCD |
| 38 | Carnitine palmitoyltransferase I deficiency, CPT1 deficiency |
| 39 | Carnitine-acylcarnitine translocase deficiency, CACT deficiency |
| **Non-MS/MS screened disorders** | |
| 40 | Glucose-6-phosphate dehydrogenase deficiency |
| 41 | Congenital Hypothyroidism, CH |
| 42 | Hereditary fructose intolerance, HFI |
| 43 | X-linked adrenoleukodystrophy, X-ALD |
| 44 | Mucopolysaccharidosis |
| 45 | Globoid cell leukodystrophy, GLD |
| 46 | Gaucher disease, GD |
| 47 | Fabry disease |
| 48 | Niemann-Pick disease |
| 49 | Beta-thalassemia |
| 50 | Hemophilia B，HB |
| 51 | Glycogen storage disease |
| 52 | Growth hormone deficiency, GHD |
| 53 | Vitamin D-dependent rickets |
| 54 | X-linked dominant hypophosphatemia, XLH |
| 55 | Cartilage-hair hypoplasia, CHH |
| 56 | Spinal muscular atrophy，SMA |
| 57 | Galactosemia |
| 58 | Crigler-najjar syndrome，CNS |
| 59 | Progressive familial intrahepatic cholestasis, PFIC |
| 60 | Nephroblastoma, NB |
| 61 | Retinoblastoma, RB |
| 62 | Non-syndromic hearing loss, NSHL |
| 63 | Mitochondrial encephalomyopathy, lactic acidosis and stroke-like episodes，MELAS |
| 64 | Severe combined immune deficiency, SCID |
| 65 | Autoimmune enteropathy, AIE |
| 66 | X-linked recessive chronic granulomatous disease, X-CGD |
| 67 | X-linked agammaglobulinemia, XLA |
| 68 | Wiskott-Aldrich syndrome, WAS |
| 69 | Dihydropyrimidine dehydrogenase deficiency |
| 70 | Purine drug toxicity |
| 71 | Inborn errors bileacid synthesis, IEBAS |
| 72 | Wilson disease, WD |
| 73 | Congenial disorders od glycosylation, CGD |
| 74 | Sitosterolemia |

Table S3. Genetic analysis and clinical data of NBGS negative/ C-NBS true positive newborns

| Cases | Disorders | Gene | NBGS panel | IEM panel | Biochemical results | BW GA  (grams) (weeks) | |
| --- | --- | --- | --- | --- | --- | --- | --- |
| 1 | Phenylalanine hydroxylase deficiency | *PAH* | c.688G>A | c.688G>A  c.617A>G (VUS) | Phe=153, PHE/TYR=1.66 | 2850 | 38+0 |
| 2 | Phenylalanine hydroxylase deficiency | *PAH* |  | c.463C>T(VUS) c.526C>T | Phe=121, PHE/TYR=1.9 | 3520 | 40+0 |
| 3 | Phenylalanine hydroxylase deficiency | *PAH* | c.1174T>A | c.739G>T(VUS) c.1174T>A | Phe=138, PHE/TYR=1.6 | 3600 | 39+2 |
| 4 | Congenital hypothyroidism | *DUOX2* | c.1588A>T | / | TSH=55 | 3530 | 38+2 |
| 5 | Congenital hypothyroidism | *DUOX2* | ND | / | TSH=14.8 | 3570 | 39+5 |
| 6 | Congenital hypothyroidism | *DUOX2* | ND | / | TSH=34 | 2840 | 37+1 |
| 7 | Congenital hypothyroidism | *DUOX2* | ND | / | TSH=59 | 3460 | 39+2 |
| 8 | Primary systemic carnitine deficiency | *SLC22A5* | c.1400C>G | c.1400C>G  c.780 797del | C0=8.20 | 3100 | 38+6 |
| 9 | Primary systemic carnitine deficiency | *SLC22A5* | c.1400C>G | c.137C>T(LP)  c.1400C>G | C0=5.01 | 4190 | 40+2 |
| 10 | Primary systemic carnitine deficiency | *SLC22A5* | c.51C>G | c.51C>G  c.388G>A | C0=4.61 | 3600 | 38+5 |
| 11 | Primary systemic carnitine deficiency | *SLC22A5* | c.51C>G | c.51C>G  c.674C>T (VUS) | C0=7.57 | 3400 | 38+4 |
| 12 | Maple syrup urine disease | *BCKDHA* | c.1309_1310del | c.712G>A  c.1309_1310del | LEU+ILE+PRO-OH=2562.69，LEU=2562，VAL=914 | 4150 | 39+3 |
| 13 | Short-chain acyl-CoA dehydrogenase deficiency | *ACADS* | 682G>A | c.209A>C(VUS) c.682G>A |  | 3400 | 38+6 |
| 14 | Short-chain acyl-CoA dehydrogenase deficiency | *ACADS* | c.682G>A | c.682G>A  c.360+1G>A (VUS) |  | 3250 | 38+4 |
| 15 | Very long-chain acyl-CoA dehydrogenase deficiency | *ACADVL* | ND | c.919A>T  c.1054A>G | Multiple long chain acylcarnitine elevated | 3640 | 39+5 |
| 16 | Tyrosinemia | *TAT* | ND | c.890G>T  c.480+1G>A | TYR=625.60 | 3700 | 39+4 |
| 17 | Ornithine transcarbamylase deficiency | *OTC* | ND | c.931G>A |  | 3600 | 39+0 |

(“ND” indicates the newborns identified no mutation by NBGS; “/” indicates the newborns were not detected by other genetic analysis; BW, birth weight (in grams); GA, gestational age (in weeks))

Table S4. Genetic analysis and clinical data of NBGS negative/ primary C-NBS positive newborns in the lost to followed-up subgroup

| NO. | Biochemical result | BW  (g) | GA  (weeks) | Conditions | NBGS panel | IEM panel  gene (mutations) |
| --- | --- | --- | --- | --- | --- | --- |
| 1 | Cit=2.78  Recheck | 2900 | 38+6 | Carbamoyl phosphate syntetase I deficiency | No mutation | *CPS1*  (c.3405-1G>T/ c.3075T>A) |
| 2 | C50H=0.89 | 2350 | 39+5 | 2-methylbutyryl-CoA dehydrogenase deficiency | No mutation | *ACADSB*  (c.1165A>G/ c.1165A>G) |

Table S5. NBGS result of 29 C-NBS false positive and C-NBS negative newborns with variants related to non-metabolic disorders

| NO. | The category of diseases | gene | Genotype | Cases |
| --- | --- | --- | --- | --- |
| 1 | Mitochondrial non-syndrome  hearing loss | *MT-RNR1* | 4*[1496C>T]; 13*[ 1557A>G]; | 17 |
| 2 | Hearing loss | *GJB2* | [c.235delC/ c.176_191del] | 1 |
| 3 | ΔMitochondrial encephalomyopathy | *MT-TL1* | 6*[3224A>G] | 6 |
| 4 | ΔHepatolenticular degeneration | *ATP7B* | [c.3859G>A/ c.3859G>A]; [c.2333G>A/ c.3532A>G] | 2 |
| 5 | ΔPoor metabolism of thiopurines 2 | *NUDT15* | AD: 2*[c.37_42del] | 2 |
| 6 | Δ X-linked dominant hypophosphatemic rickets/osteomalacia | *PHEX* | [c.1482dup/ c.1482dup] | 1 |
| Total |  |  |  | 29 |

(AD: autosomal dominant; “Δ” indicates the diseases not included in previous newborn screening but screened positive by NBGS panel)

Table S6. The list of 77 genes in which one or more variants were detected

| *SLC22A5* | *DUOXA2* | *MAT1A* | *ACAT1* | *RMRP* | *AGL* | *NPC1* |
| --- | --- | --- | --- | --- | --- | --- |
| *DUOX2* | *SLC25A13* | *GBA* | *SMPD1* | *PCCB* | *ETFA* | *UGT1A1* |
| *GJB2* | *PTS* | *BCKDHA* | *SLC25A15* | *HLCS* | *GCH1* | *GALNS* |
| *ACADS* | *ABCG5* | *GAA* | *GALT* | *ASL* | *HADHB* | *IDUA* |
| *ATP7B* | *MMACHC* | *GALE* | *BTD* | *CBS* | *HPD* | *LMBRD1* |
| *PAH* | *ACADM* | *DPYD* | *GCDH* | *IL7R* | *MLYCD* | *MMAB* |
| *SLC26A4* | *G6PC* | *GALC* | *MTR* | *ABCD4* | *MMAA* | *NAGLU* |
| *ASS1* | *GJB3* | *PMM2* | *TPO* | *MTHFR* | *QDPR* | *NAGS* |
| *MMUT* | *MCCC1* | *BCKDHB* | *ALDOB* | *NPC2* | *RAG1* | *OAT* |
| *HBB* | *CPT2* | *CYP27B1* | *ADA* | *PYGL* | *SLC37A4* | *PCBD1* |
| *ACADVL* | *ETFDH* | *SLC25A20* | *ARG1* | *TG* | *HMGCS2* | *TSHR* |

Table S7. The list of 5 genes associated with susceptibility to certain conditions of carriers

| Gene | OMIM-ID | Inheritance | OMIM phenotype ID | OMIM-Phenotype |
| --- | --- | --- | --- | --- |
| *MTR* | 156570 | AR | 601634 | {Neural tube defects, folate-sensitive, susceptibility to} |
| *TG* | 188450 | . | 608175 | {Autoimmune thyroid disease, susceptibility to, 3} |
| *GBA* | 606463 | AD \| AD,Mu | 127750 \| 168600 | {Lewy body dementia, susceptibility to} \|  {Parkinson disease, late-onset, susceptibility to} |
| *MTHFR* | 607093 | AR \|  AD \|  AD | 601634 \| 181500 \| 188050 | {Neural tube defects, susceptibility to} \|  {Schizophrenia, susceptibility to} \|  {Thromboembolism, susceptibility to} |
| *CPT2* | 600650 | AD,AR | 614212 | {Encephalopathy, acute, infection-induced, 4, susceptibility to} |

(The “.” represents the inheritance is unclear)
